# Conditioning invasive bigheaded carps (*Hypophthalmichthys molitrix* and *H. nobilis*)to enhance the efficacy of acoustic and CO_2_ deterrents

**DOI:** 10.1371/journal.pone.0320395

**Published:** 2025-05-19

**Authors:** Jack A. Culotta, Marie L. Ervin, Brooke J. Vetter, Allen F. Mensinger

**Affiliations:** 1 Biology Department, University of Minnesota, Duluth, Minnesota, United States of America; 2 Department of Biology, University of St. Thomas, St. Paul, Minnesota, United States of America; PLOS: Public Library of Science, BASS Division, 48 Watson Street, UNITED KINGDOM OF GREAT BRITAIN AND NORTHERN IRELAND, Aberdeen, AB25 2QL

## Abstract

Invasive bigheaded carps (*Hypophthalmichthys molitrix* and *H. nobilis*) have caused substantial ecological and economic damage throughout the Mississippi River Basin and expanded their range threatening the Laurentian Great Lakes. Broadband acoustic deterrents have shown promise in repelling carp and are currently being assessed in navigational lock chambers on the Mississippi River. These nonphysical deterrents permit vessel navigation while reducing carp passage. However, no single deterrent is 100% effective and fish may habituate to the sound after repeated playback. Carp exhibit aversive behaviors to carbon dioxide, which suggests combining these two stimuli into one deterrent system could extend the effective duration of sound and reduce the frequency of carbon dioxide (${CO}_{2}$) application. We conditioned bigheaded carps to associate broadband sound from outboard boat motors (0.06–5 kHz, ~150 dB re. 1 μPa) with ${CO}_{2}$ application (~35,000 ppm) in small (80 L) and large (3475 L) two-choice shuttle tanks. We compared negative phonotaxis responses over one to four weeks between fish conditioned with sound and ${CO}_{2}$, sound and air, or sound alone. Similar ${CO}_{2}$ avoidance thresholds were found across tank sizes and species. Conditioning treatment did not affect time to leave the sound chamber, confirming sound alone remains a deterrent for all fish. Carp conditioned with ${CO}_{2}$ took longer to return to the sound chamber than control treatments. Control fish were closer to the speaker during playback than during the pre-sound period, while fish conditioned with ${CO}_{2}$ were not significantly closer. Conditioning paradigms may extend the effective duration of nonphysical deterrents for bigheaded carps. Conditioning with ${CO}_{2}$ may also increase proactive flight-responses over reactive freeze-responses. Findings could be applied to increase nonphysical barrier effectiveness at locks along the Mississippi River and help protect the Laurentian Great Lakes from invasion.

## Introduction

Invasive bigheaded carps (*Hypophthalmichthys molitrix* and *H. nobilis*) have caused substantial ecological and economic damage throughout the Mississippi River Basin and continue to expand their range, thereby threatening colonization of the Laurentian Great Lakes [1–3]. Broadband acoustic deterrents have shown promise in repelling carp and are currently being assessed in navigational locks on the Mississippi River. These nonphysical deterrents permit vessel navigation while reducing carp passage. However, no single deterrent was 100% effective, and fish may habituate to the sound after repeated playback [4]. For example, broadband sound alone may not be an effective deterrent to carp passage through navigational locks, indicating that multi-modal sensory information may be more effective [5]. The bigheaded carps hybridize in the wild [6], and share invasion histories and range expansion routes [7], suggesting that they be managed together. After invasive species establish reproductive populations, extirpation is nearly impossible, so prevention is the most efficient and cost effective control method [8]. Carp barrier technology aims to prevent further range expansion [9,10], with the ultimate goal of preventing the passage of bigheaded carps, while not affecting the migration of native fishes [11].

The Mississippi River Basin is an important artery for commercial vessels with numerous dams that regulate water levels and flow to improve navigation. Most dams regulate water flows with integrated gates and provide nearly continuous physical barriers to fishes and only during high flow events (i.e. floods), when the gates are fully opened, dams are overtopped or adjacent areas are flooded, are fish likely to cross [12]. However, adjacent navigation locks routinely operate throughout the shipping season allowing fish passage past the dam. These locks may serve as bottlenecks to upstream migration and are being targeted for carp deterrents either in the chamber or the downstream approach. Due to the commercial importance of the waterways, it is imperative that these nonphysical deterrents do not affect vessel navigation. However, no single nonphysical barrier has been proven to block 100% of fish [13], and therefore combining multiple aversive stimuli into integrated deterrent systems could improve overall deterrence.

Sound and carbon dioxide (${CO}_{2}$) function as behavioral deterrents for bigheaded carps. Bigheaded carp have demonstrated consistent negative phonotaxis in response to broadband sound, while habituation occurred to pure tone stimuli [14,15]. Broadband sound was further shown to be effective at preventing passage through narrow channels [16]. As ostariophysans, bigheaded carps possess Weberian ossicles that provide a connection between the inner ear and swim bladder and expand the upper frequency range to 5 kHz [17,18]. Many native fishes, such as lake sturgeon (*Acipenser fulvescens*), paddlefish (*Polyodon spathula*), and largemouth bass (*Micropterus salmoides*) are non-ostariophysans and are therefore unlikely to detect sound frequencies beyond 1 kHz [19]. Furthermore, when exposed to broadband outboard motor sound, native fish (including ostariophysans and non-ostariophysans) exhibited minimal, or no, negative phonotaxis. In contrast, bigheaded carp show consistent negative phonotaxis [20]. Therefore, acoustical deterrents in the 2–4 kHz range are likely to deter ostariophysian fishes, but not be detectable by native non-ostariophysan species in the Mississippi River basin.

Additionally, all fish have ${CO}_{2}$ receptors on their gills [21], and most fishes exhibit negative chemotaxis to ${CO}_{2}$ [11,22–24] suggesting that elevated ${CO}_{2}$ levels will deter carp movement. Bighead carp have been found to consistently relocate away from ${CO}_{2}$ injection sites [25–27]. Carbon dioxide is being evaluated as a potential carp deterrent specifically for application at lock and dam systems [28], and see Suski for a review of deterrents in carp]. Common carp (*Cyprinus carpio*) exit a navigational lock more quickly in a forced current and ${CO}_{2}$ injection condition compared to a null treatment, although they exited more slowly than in a forced current treatment alone [29]. Further, injection systems for ${CO}_{2}$ can be reasonably optimized to reach thresholds for behavioral avoidance in locks [30]. However, ${CO}_{2}$ does not specifically target bigheaded carp and may be detrimental to the environment, so it would be desirable to limit its application frequency. Combining ${CO}_{2}$ with another deterrent could minimize the treatment rates. Therefore, if bigheaded carp could be classically conditioned to associate sound with ${CO}_{2}$ release, it may be possible to increase the effectiveness of both deterrents, by extending the effective duration of acoustic deterrents, and lessening the frequency of ${CO}_{2}$ deployment. Common carp can be classically conditioned to associate sound with a food reward with responses persisting for at least four months, suggesting the potential for acoustic conditioning in this family [31].

Our aim is to determine whether bigheaded carps can be conditioned to associate ${CO}_{2}$ with sound to increase the efficacy of nonphysical deterrents, and to evalate the effective duration of conditioning. While bigheaded carp show strong aversion to both ${CO}_{2}$ and acoustic stimuli [32], ours is the first study to evaluate behavioral responses of conditioned and unconditioned bigheaded carps with the combination of acoustic and ${CO}_{2}$ stimuli. The interactive effects of ${CO}_{2}$ and sound on fish behavior have not been extensively previously studied. However, in European sea bass (*Dicentrarchus labrax*), and damselfish (*Pomacentrus wardi*), interactive effects of elevated ${CO}_{2}$ and anthropogenic noise on predator-prey dynamics were not found [33,34].

## Results

### ${CO}_{2}$ threshold

**Fig 1:**
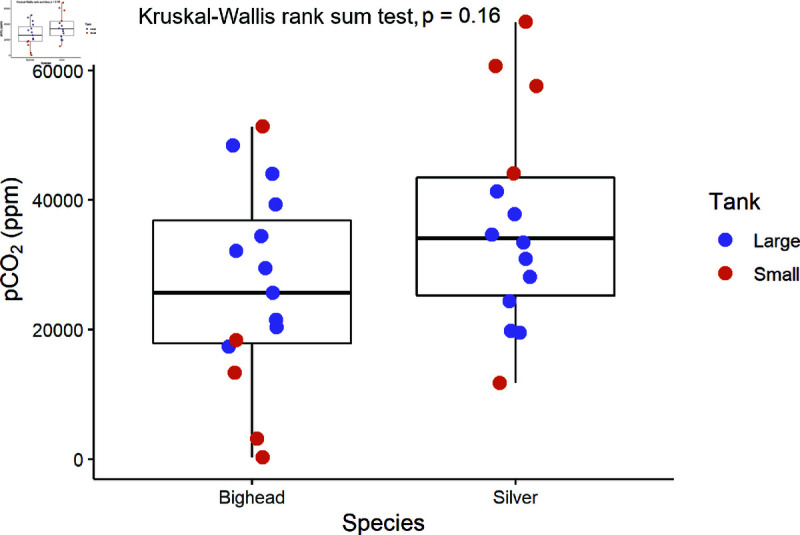
<figuretext>${CO}_{2}$ threshold.</figuretext> Boxplots indicate the median p${CO}_{2}$ concentration threshold that resulted in fish leaving the ${CO}_{2}$ side of the shuttle tank. Boxplots display the median ± quartiles with whiskers extending to 1.5 times IQR. Data points represent individual trials from small (red) and large (blue) tanks. There was no significant difference in threshold between species (Kruskal-Wallis rank sum test, ${\text{X}}^{2}=2.07$, *p* = 0.15).

Initial ${CO}_{2}$ injection in one half of the shuttle tank stimulated fish movement between both sides of the tank before eventually causing fish to permanently leave the ${CO}_{2}$ side or lose equilibrium. We determined threshold ${CO}_{2}$ concentrations when the majority of the school (3 or more fish) or the single bighead carp (large tank only) exited the ${CO}_{2}$ chamber. In the small shuttle tank, schools of unconditioned bighead carp (*n* = 5 schools; *n* = 5 individuals per school) exhibited ${CO}_{2}$ avoidance at 17,278 ± 9,119 ppm (*n* = 5 schools, mean ± SE, Table 1). Schools of silver carp (*n* = 5 schools; *n* = 5 individuals per school) exhibited avoidance at 48,360 ± 9,901 ppm. In the large shuttle tank, individual bighead carp (*n* = 10) exhibited ${CO}_{2}$ avoidance at 31,272 ± 3,293 ppm, while schools of silver carp (*n* = 10 schools; *n* = 5 individuals per school) exhibited avoidance at 28,591 ± 2,429 ppm. There was no significant difference in dissolved ${CO}_{2}$ thresholds between tank size (Kruskal-Wallis rank sum test, ${\text{X}}^{2}=0.00,$ *p* = 1.00) or species (Kruskal-Wallis rank sum test, ${\text{X}}^{2}=2.07$, *p* = 0.15, Fig 1).

**Table 1:** ${CO}_{2}$ avoidance thresholds in two experimental shuttle tanks

Tank	Species	TA (ppm)	Temp (°C)	${CO}_{2}$ (ppm)
Small	Bighead	169 ± 3	20.5 ± 0.1	17,278 ± 9,119
Small	Silver	165 ± 2	20.7 ± 0.1	48,360 ± 9,901
Large	Bighead	242 ± 2	17.3 ± 0.2	31,273 ± 3,293
Large	Silver	227 ± 3	16.1 ± 0.4	28,591 ± 2,429

TA, total alkalinity, temperature, and dissolved ${CO}_{2}$. Data represent mean ± 1 SE.

### Exit time

#### Small tank

**Fig 2:**
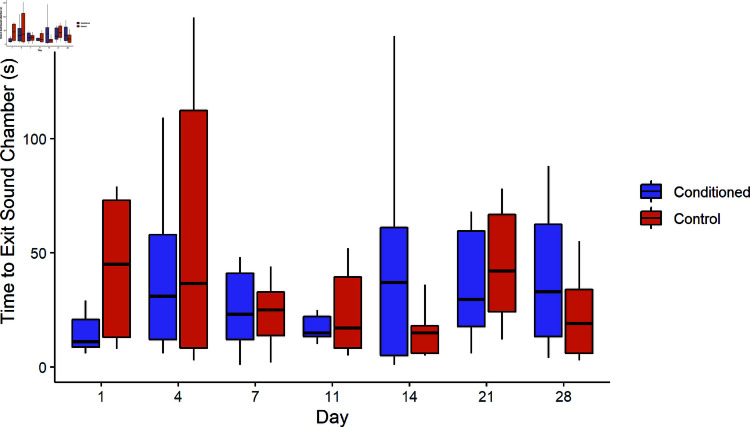
Exit time in the small tank. Box plots indicate the median time that unconditioned (blue) or ${CO}_{2}$ and sound conditioned schools (red) exited the small sound chamber after sound onset. Boxplots include the median ± quartiles for each treatment. Whiskers extend to 1.5 times IQR. The difference between conditioned and unconditioned schools on the first day was not significant (Welch Two Sample t-test, df = 1.95, *t* = 1.95, *p* = 0.07).

Sound initiation elicited negative phonotaxis in both conditioned and unconditioned schools, as the schools exited the sound chamber and swam to the opposite shuttle tank chamber. On the first day of negative phonotaxis trials, there was no difference in the time conditioned schools of silver (*n* = 10 schools; *n* = 5 individuals per school) and bighead (*n* = 10 schools; *n* = 5 individuals per school) carp left the sound chamber (19 ± 17 s), compared to unconditioned schools (42 ± 31 s) (Two Sample t-test, df = 1.95, *t* = 1.95, *p* = 0.07). All conditioned carp exhibited negative phonotaxis by shuttling out of the sound chamber, however, two unconditioned schools did not exit the sound chamber within the five minutes of acoustic playback. Sound had a significant effect on time until the first exit (mixed ANOVA, ${\text{X}}^{2}=10.32$, df = 1, *p* < 0.001). There was a significant interaction between conditioning treatment and day since conditioning (Mixed ANOVA, ${\text{X}}^{2}=6.60$, df = 1, *p* = 0.01). There were no differences in exit time for conditioning treatment on later sound presentations (Fig 2).

#### Large tank

**Fig 3:**
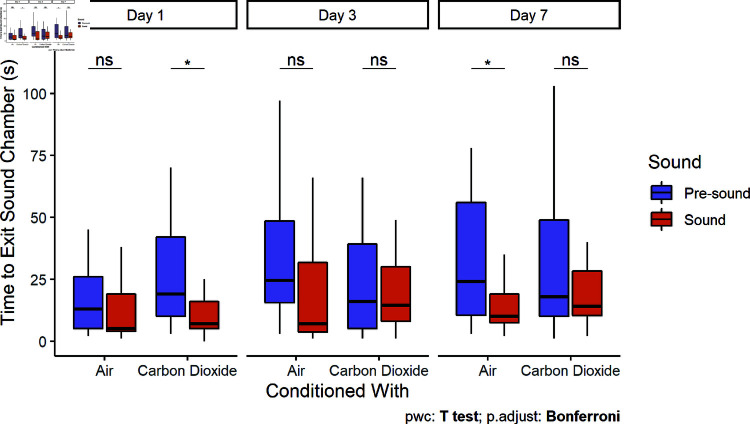
Exit time in the large tank. Box plots indicate the median time that bighead carp conditioned with air and sound (blue) or ${CO}_{2}$ and sound (red) exited the large sound chamber after sound onset. Boxplots display the median ± quartiles with whiskers extending to 1.5 times IQR. Bighead carp conditioned with ${CO}_{2}$ exited the sound chamber significantly faster when the sound stimulus was playing than during the pre-sound period on the first day (Paired t-test, t = 2.72, df = 20, p = 0.013).

Bighead carp conditioned with ${CO}_{2}$ exited the sound chamber significantly faster when the sound stimulus was playing (11 ± 8 s) than during the pre-sound period (31 ± 29 s) on the first day (Paired t-test, *t* = 2.72, df = 20, *p* = 0.013, Fig 3), and on the seventh day (27 ± 29, 44 ± 48, Paired t-test, *t* = 2.18, df = 14, *p* = 0.047). There were not any other significant differences in exit time. In a mixed ANOVA of exit time, two predictors were highly significant: sound (${\text{X}}^{2}=11.43$, df = 1, *p* < 0.001) and day (${\text{X}}^{2}=10.62$, df = 2, *p* = 0.005). The three-way interaction of conditioning treatment, sound, and day was not significant ($X^{2}=5.16$, df = 2, *p* = 0.076).

### Return time

#### Small tank

**Fig 4:**
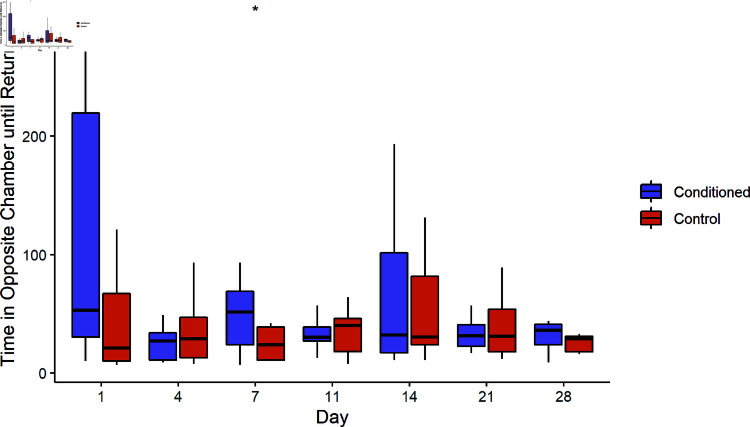
Return time in the small tank. Box plots indicate the median time that conditioned carp (red) and unconditioned carp (blue) spent in the opposite chamber after sound onset. Boxplots display the median ± quartiles with whiskers extending to 1.5 times IQR. * indicates significantly different medians (Welch Two Sample t-test, df = -2.34, t = -2.34, p = 0.04).

On the first day of phonotaxis trials, there was no significant difference in the time conditioned schools spent before returning to the chamber containing the sound source (115 ± 112 s) compared to unconditioned schools (44 ± 42 s, Welch Two Sample t-test, df = −1.85, t = −1.85, p = 0.09). On day seven, conditioned schools spent a significantly longer time (47 ± 28 s) than unconditioned schools (24 ± 13 s; Welch Two Sample t-test, df = -2.34, t = −2.34, p = 0.04, Fig 4) away from the sound, however there was no significant difference for any other day.

Conditioning treatment alone did not have a significant effect on return time to the sound chamber (Mixed ANOVA, $X^{2}=3.03$, p = 0.082), however the acoustic signal ($X^{2}=8.22$, p = 0.004), and the number of days since conditioning had a significant effect on return time ($X^{2}=4.09$, p = 0.043). There was also a significant three-way interaction between conditioning treatment, presence of sound, and the number of days since trial onset ($X^{2}=4.24$, p = 0.039).

#### Large tank

**Fig 5:**
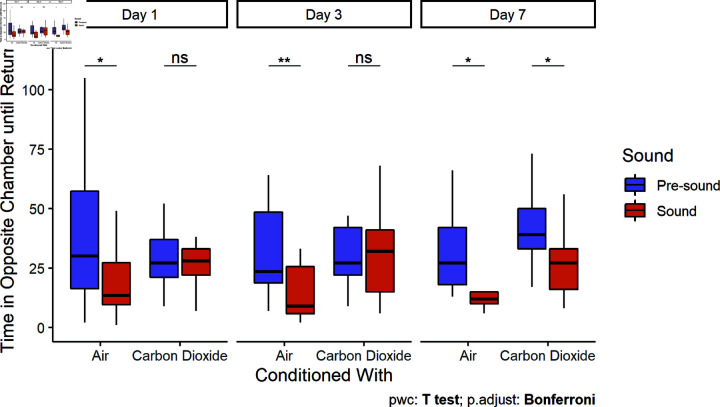
Return time in the large tank. Boxplots include the median ± quartiles for each period. Whiskers extend to 1.5 times IQR. Control carp returned to the sound chamber more quickly than they had during the pre-sound period for the first (Paired t-test, t = 2.64, df = 20, p = 0.016), second (Paired t-test, t = 3.01, df = 16, p = 0.009, and third days (Paired t-test, t = 2.91, df = 9, p = 0.020). Carp conditioned with ${CO}_{2}$ did not return significantly faster until the third day (Paired t-test, t = 2.30, df = 16, p = 0.035).

Control bighead carp returned to the sound chamber significantly faster when the sound stimulus was playing than during the pre-sound period on the first (Paired t-test, t = 2.64, df = 20, p = 0.016), second (Paired t-test, t = 3.01, df = 16, p = 0.009, and third days (Paired t-test, t = 2.91, df = 9, p = 0.020, Fig 5). In contrast, bighead carp conditioned with ${CO}_{2}$ returned to the sound chamber as quickly as they had during the pre-sound period the first and third day after conditioning. However, one week after conditioning, bighead carp conditioned with ${CO}_{2}$ began returning to the sound chamber more quickly than they had during the pre-sound period (Paired t-test, t = 2.30, df = 16, p = 0.035), exhibiting similar behavior to control carp.

In the large tank the acoustic signal significantly affected time to return (Mixed ANOVA, $X^{2}=24.55$, p < 0.001), as did the trial day (Mixed ANOVA, $X^{2}=6.97$, p = 0.031). Although there was not a significant three-way interaction as in the small tank, the interaction of sound with conditioning treatment was significant (Mixed ANOVA, $X^{2}=5.67$, p = 0.017).

### Flight and freeze responses

**Fig 6:**
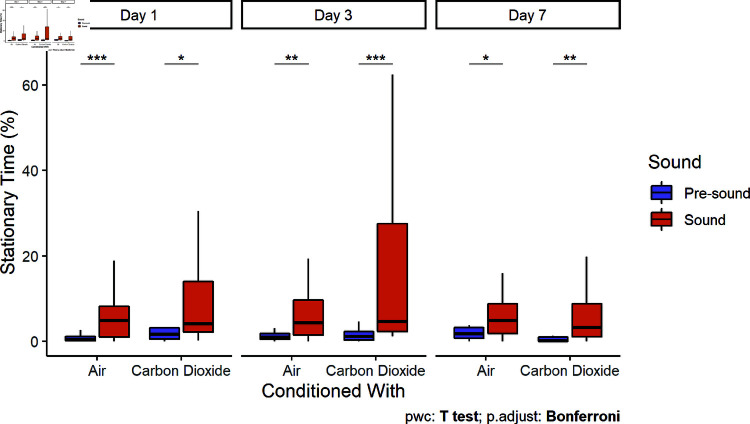
Stationary time (large tank). Bighead carp from both conditioning treatments exhibited freeze-responses to sound playback. Boxplots include median ± quartiles for each conditioning treatment and sound stimulus. Whiskers extend to 1.5 times IQR. Points indicate responses outside of the IQR.

**Fig 7:**
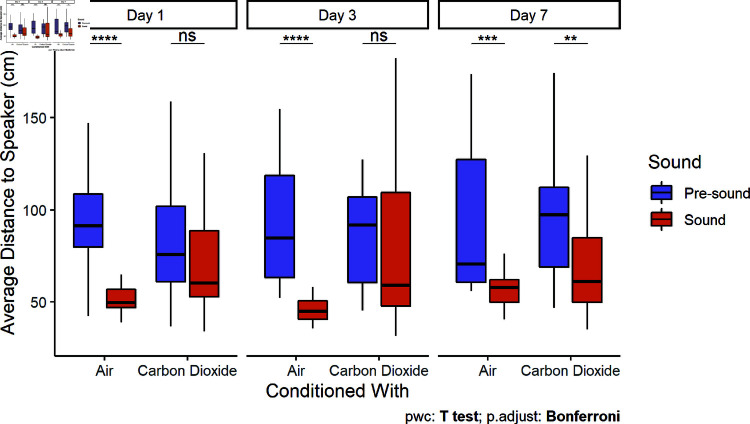
Distance to the speaker (large tank). Boxplots include the median ± quartiles for each period. Whiskers extend to 1.5 times IQR. Carp conditioned with air remained significantly closer to the active speaker on the first (Paired t-test, t = 8.89, df = 26, p < 0.0001), third (Paired t-test, t = 6.35, df = 25, p < 0.0001), and seventh days (Paired t-test, t = 4.64, df = 25, p < 0.0001). In contrast, carp conditioned with ${CO}_{2}$ did not remain significantly closer to the speaker during playback until the seventh day (Paired t-test, t = 3.51, df = 29, p = 0.001).

During the pre-sound period, bighead carp swam in a figure-eight pattern through both chambers and rarely spent any time stationary. During the initial sound presentation, fish in both the unconditioned and conditioned groups exhibited freeze-responses (i.e., to stop swimming and remain stationary (Fig 6). However, bighead carp conditioned with ${CO}_{2}$ were more likely to exhibit flight-responses (i.e., movements away from the acoustic stimulus). This was quantified as control fish had closer (Two Sample t-tests, df = 27,25,25, t = 8.89, 6.35, 4.64, p < 0.0001) average distances to the speaker during playback (54 ± 21 SD, 57 ± 30, 67 ± 41 cm) compared to the pre-sound period (97 ± 26, 95 ± 35, 98 ± 44 cm) for all three days, whereas those conditioned with ${CO}_{2}$ were not significantly closer (Two Sample t-tests, df = 28,29, t = 1.42, 1.63, p = 0.17, 0.11) to the speaker on the first and third day. By the seventh day after conditioning, bighead carp conditioned with ${CO}_{2}$ exhibited similar behavior to control fish by staying closer (69 ± 25 cm) to the speaker than they had during the pre-sound period (95 ± 30 cm; Two Sample t-test, df = 29, t = 3.51, p = 0.001; Fig 7).

**Fig 8:**
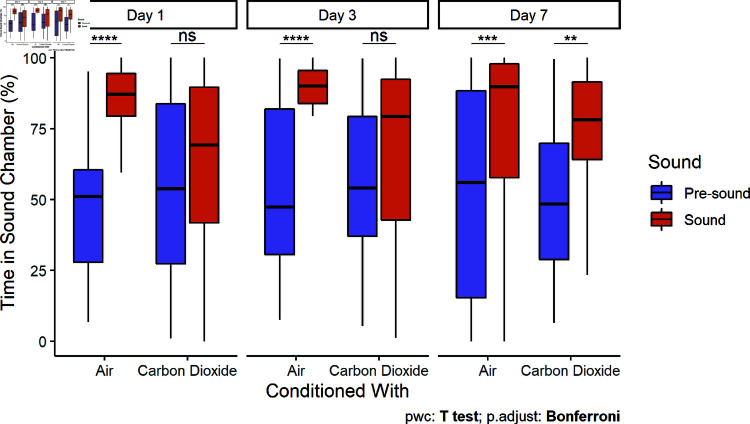
Duration in sound chamber (large tank). Boxplots include the median ± 25% and 75% quartiles for each period. Whiskers extend to 1.5 times IQR. Control carp spent significantly more time in the sound chamber during playback compared to the pre-sound period on the first (Paired t-test, t = -8.36, df = 28, p < 0.0001), third (Paired t-test, t = -5.59, df = 26, p < 0.0001), and seventh days (Paired t-test, t = -3.84, df = 29, p < .001). Carp conditioned with ${CO}_{2}$ did not spend significantly more time in the sound chamber on the first and third days, but did on the seventh day (Paired t-test, t = -3.58, df = 29, p = .001).

Control carp spent a larger proportion of time in the sound chamber than they had during the pre-sound period for all trial days. Carp conditioned with ${CO}_{2}$ did not spend significantly more time in the sound chamber until the seventh day after conditioning (Fig 8).

## Discussion

We are the first to examine the effects of conditioning carp with ${CO}_{2}$ and sound. Bigheaded carps avoided ${CO}_{2}$ at a reproducible threshold consistent with a previous studies [27,35]. Compared to control fish, aversive behaviors to the acoustic stimulus were stronger and lasted longer over repeated unpaired exposures across multiple days in fish conditioned with sound and ${CO}_{2}$. Control fish were more likely to remain closer to the speaker during playback than carp conditioned with ${CO}_{2}$, which exhibited negative phonotaxis.

While ${CO}_{2}$ is an effective deterrent, environmental release and effects on native species are problematic. Therefore, minimizing its usage would appeal to management agencies. As deterrent systems will need to prevent invasive species range expansion indefinitely, a conditioning paradigm may lessen the frequency of application and long-term costs. We conditioned bigheaded carps with sound and dissolved ${CO}_{2}$ and examined their response to sound alone. We found intermittent ${CO}_{2}$ application combined with acoustic deterrence to be an effective strategy as it will reduce the need for ${CO}_{2}$ inputs, minimizing effects on native species, while extending the efficacy of acoustic deterrents.

Multiple species share similar ${CO}_{2}$ avoidance thresholds as those we measured in bigheaded carp [24]. Previous behavioral studies of bigheaded carps demonstrated ${CO}_{2}$ avoidance at a concentration ranging from ~30,000 to 40,000 ppm [27,35] and showed bighead carp increase lateral movements between flumes injected with ${CO}_{2}$, searching for areas with lower ${CO}_{2}$ [26]. A similar response to ${CO}_{2}$ for both species could allow ${CO}_{2}$ deterrents to manage both species successfully.

The acoustic stimulus facilitated movements away from the speaker in bigheaded carp conditioned with either air or ${CO}_{2}$. The sound needs to continue to promote negative phonotaxis in unconditioned fish as in a field setting, all fish near the lock need to be deterred regardless of whether they have experienced intermittent carbon dioxide conditioning. The conditioned fish were more averse to returning to the sound chamber than control fish demonstrating the additional benefit of combining the deterrents. The conditioning effect diminished by day seven, suggesting that more extended conditioning may be necessary.

Small tanks create complex sound environments [36–38]. Previous acoustic deterrent experiments on bigheaded carp used rectangular outdoor ponds to move fish from one end to the other or block passage through a narrow channel [15,16]. The shuttle tank incorporated two circular tanks connected by a narrow channel with the speaker located centrally in each tank. While excellent for maintaining separate ${CO}_{2}$ environments, the soundscape appeared challenging to the fish, and the lack of clear sound gradients in the sound chamber appeared to confuse the fish or delay escape from the chamber. Our ongoing experiments using a model lock and dam have in contrast shown clear and directed movement away from the sound due to clear sound gradients. This future work should help address comparisons to real-world conditions such as water flow and motivation to travel upstream, either for food or reproduction. While the shuttle tanks and carbon dioxide injection system provided precise mechanisms for accurately and quickly controlling the carbon dioxide levels in one side of the arena, the complex acoustic field of the shallow, circular tanks made it challenging to assess the combined effects of the two deterrents.

In the large shuttle tank, the side opposite the active speaker offered both a sound pressure and particle acceleration “refuge” with lower intensities. However, the sound chamber and path leading to the channel was more complicated. It was somewhat surprising that fish did not immediately seek the channel and quieter side. Previous experiments in large rectangular outdoor ponds showed clear paths away from the sound source. In both shuttle tanks, however, fish often seemed to have trouble locating the channel. This was especially apparent in fishes that had the speaker between them and the channel in the circular section.

Controlled experiments determine how fish behavior is altered by certain stimuli and association paradigms, as well as assess cognitive processes like retention and habituation. Behavioral studies using classical or operant conditioning provide a more sensitive assessment of fish hearing than electrophysiological experiments alone [39]. Further, acoustic behavioral studies are useful to determine not only what sounds fish detect but also what they respond to, especially when using complex signals, like broadband sound [40].

Fish behavioral responses to the onset of a sudden stimulus can be proactive (fight or flight-responses) or reactive (freeze-responses) [41]. A reactive, low movement strategy was often chosen by fish in both treatment groups (Fig 6). Previous studies in shuttle box systems have observed freeze-responses to aversive stimuli, like ${CO}_{2}$ [42,43]. In our study, carp conditioned with ${CO}_{2}$ were more likely to flee (i.e., leave the sound chamber) than exhibit freeze-responses (i.e., remain in place near the speaker), particularly in the first two days (Fig 7). In contrast, common carp were found to exhibit increased encamped behavior when exposed to ${CO}_{2}$ in the field [29]. With increasing duration of a stressor, rainbow trout (*Oncorhynchus mykiss*) tended to transition from proactive to reactive coping styles [44]. If a fish is not able to escape a chronic stressor, it may be more adaptive to switch to an energy saving strategy than continuing to act proactively by attempting to flee. Pairings of sound and ${CO}_{2}$ may encourage a conditioned response to behave proactively to sound by actively leaving an area, but fish will respond increasingly reactively over longer exposures. However, the freeze-response alone may be a sufficient response for some acoustic deterrents in a riverine system, as deterrents need only to prevent swimming upstream, and the downstream current would push carp away from passage opportunities. The maintenance of fleeing behavior may help clear already occupied lock chambers, whereas it would also be sufficient if freeze-responses can be achieved prior to entry of the lock chamber.

Wild carp challenging an acoustic deterrent in the field will be presented multiple opportunities to cross upstream per day [45]. In the small tank, the responses of conditioned carp attenuated to resemble those of unconditioned fish one week after conditioning. In the large tank, sound was presented three times each testing day to determine whether habituation occurs with multiple daily exposures over one week. Carp conditioned with ${CO}_{2}$ generally exhibited more aversion to sound for the first and third day compared to control carp, and this effect diminished by the seventh day. Reapplication of carbon dioxide will likely need to occur at least weekly to prevent habituation from reducing deterrent efficacy. Alternatively, the conditioning period may need to be extended. Future studies will be necessary to determine how reinforcement with ${CO}_{2}$ affects habituation over time.

Bigheaded carps present a serious ecological threat to riverine systems and will require innovative deterrent systems to prevent their spread. In the lower Illinois River, these carps account for over 70% of all fish biomass [46]. Multi-modal deterrents have been proposed to increase efficacy, and as such two modalities were assessed here. Further, a conditioning paradigm can be employed to reduce necessary chemical inputs and minimize effects on native species, particularly of ${CO}_{2}$. This study will inform river management for nonphysical barriers at lock and dam systems across the invasive range of bigheaded carps.

In conclusion, we show that bigheaded carp can be conditioned with sound and ${CO}_{2}$ over two days, that this association can be retained over multiple unpaired sound exposures, and that conditioned responses may last up to one week. Prior conditioning with ${CO}_{2}$ increases avoidance of acoustic signals, possibly by encouraging proactive avoidance strategies (i.e., flight-responses) over reactive ones (i.e., freeze-responses). Future studies should assess whether reinforcement with intermittent pairing of ${CO}_{2}$ maintains or increases avoidance responses to acoustic signals. Future research will need to confirm whether a conditioning paradigm with sound and ${CO}_{2}$ yields similar results in the field.

## Materials and methods

### Animal husbandry

The experiments were approved by the University of Minnesota Institutional Animal Care and Use Committee (Protocol No. 2103-38930A). Silver and bighead carp were obtained from the US Geological Survey (USGS) Upper Midwest Environmental Sciences Center in La Crosse, WI and transported to the Minnesota Aquatic Invasive Species Research Center (MAISRC) containment lab in St. Paul, MN. Two size classes of fish were used as experiments were held across two different tank sizes. Silver carp averaged 61 ± 1 mm total length (TL) for the small tank and 68 ± 16 mm TL for the large tank experiments. Bighead carp measured 75 ± 2 mm TL (small tank) and 210 ± 4 mm (large tank). There was not a significant difference in TL across conditioning treatments (ANOVA, F = 0.323, df = 1, p = 0.571). The small silver or bighead carp were held in monospecific schools of five fish in 45 L glass aquaria for the duration of the trials. The large bighead carp were maintained in 500 L holding tanks in groups of three to five with individuals identified by differences in total length and preexisting fin markings and notches. All fish were allowed to recover in holding tanks for one week after transport prior to trial initiation. Carp were fed equal parts Spirulina algae and dry feed (Starter Crumble; Skretting USA; Tooele, UT) and maintained on a 12-hour light cycle.

### Shuttle tanks

Two-choice shuttle tanks were used during the experiments. A small shuttle tank, consisting of two circular chambers (50 cm diameter x 25 cm depth) joined by a central rectangular channel (10 x 7 x 25 cm), was used for small bigheaded carps and designated as the “small tank” (Loligo Systems; Viborg, Denmark) [47]. The small tank was filled to a depth of 20 cm with freshwater at ambient ${CO}_{2}$ concentration (~1,500 ppm). The separate circular chambers allowed for independent control of water parameters. Each chamber received recirculated water from an adjacent buffer chamber through a continuously operating water pump (Model 300; Eheim; Deizisau, Germany). Compressed air or ${CO}_{2}$ was injected into each buffer chamber via air stones with the gas flow controlled by solenoids operated with computer software (ShuttleSoft 2.6.4; Loligo Systems). Sentix probes (Xylem Analytics; Oberbayern, Germany) in each chamber continually monitored pH. An overhead video camera (Model UI-1640SE-C-GL; Imaging and Development Systems; Stoneham, MA) continuously recorded fish position. All experiments were monitored remotely by an observer.

The standard size two choice small tank from Loligo Systems provided a proof of concept for experiments, and later trials used a scaled up larger tank size to test larger fish in more realistic conditions. The custom designed “large tank” consisted of two fiberglass circular chambers (154 cm dia x 89 cm depth) joined by a central channel (88 x 38 x 74 cm). The tank was filled to within 10 cm of the top with freshwater with ambient concentrations of ${CO}_{2}$. The tank was placed on a 1.7 cm thick rubber mat to reduce vibration. Each chamber contained air stones directly opposite the central channel through which ${CO}_{2}$ gas or air was bubbled. Two pumps (Model NH-50PX-X; Pan World; Fitchburg, MA) circulated water within the chambers in countercurrent directions to prevent mixing across the central channel. Probes were placed in both chambers to monitor instantaneous pH (Xylem Analytics). The tank bottom was lined with white marble gravel to 15 cm depth to level the chamber bottom with the central channel, to increase contrast between the fish and substate and thus improve automated video tracking, and to reduce sound propagation between the chambers. An overhead camera (Model acA1300-60gm; Basler; Ahrensburg, Germany) was mounted above the center of each chamber to record fish behavior. The two videos from each camera were joined in editing software (Wondershare Filmora 11; Wondershare Technology Group; Shenzhen, China) using the timestamp and a split screen function to represent the entire experimental tank.

In the small tank, water-resistant Bluetooth speakers (8 x 10 cm; JBL Clip 4; Harman International Industries; Stamford, CT) were placed on a 20 cm x 10 cm diameter PVC pipe with the front half of the speaker fully submerged. The sound file was played on a laptop (Model 80MK; Lenovo; Hong Kong, China) connected to the speakers via Bluetooth. In the large tank, underwater speakers (18.3 cm dia; Model UW-30; Electro-Voice; Burnsville, MN) were buried in the gravel up to the front face of the speaker. Sound files from the laptop computer were transmitted through an amplifier (Model AS-35, Accusonic; Markham, Canada) into the speaker.

### Acoustic stimulus and sound mapping

One speaker was randomly selected to produce the sound stimulus, a recording of an outboard motor (0.06 – 5 kHz) per trial. This stimulus has been previously shown to elicit negative phonotaxis in bigheaded carps [14,15,20]. Sound intensity from the active speaker was calibrated using a hydrophone (Model 96; High Tech Instruments, Long Beach, MS) positioned in the middle of the water column between the speaker and central channel. To calculate SPL, the hydrophone was connected to a PowerLab (Model ML856; AD Instruments; Castle Hill, Australia). The voltage root mean square (RMS mV) was calculated in LabChart 8 software (AD Instruments). The following equation was used to calculate SPL from RMS.

SPL in dB re. 1 $\mu$Pa=20*log(RMS)−164.4 (1)

For both tanks, sound pressure levels were calculated by measuring the RMS with a hydrophone (High Tech Inc) at multiple points evenly distributed throughout both chambers and the central connecting column. Particle acceleration (PA) in both tanks was evaluated as most non-ostariophysans only detect particle motion. PA values were measured with a triaxial accelerometer (Model W356A12/NC; PCB Piezotronics Inc; Depew, NY, United States; sensitivity, $x=10.47\;{mV/ms}^{-2}$, $y=10.35\;{mV/ms}^{-2}$, $z=10.29\;{mV/ms}^{-2}$) modified to be neutrally buoyant and connected to a signal conditioner (482C15; Piezotronics Inc) in the same locations as SPL measurements and are reported in the public dataset (44). PA was calculated by measuring the RMS for each of the three axes, conversion to magnitude vectors, and through the following equation:

$$\text{PA in dB re. 1 }ms^{-2}=20*log\sqrt{x^{2}+y^{2}+z^{2}}$$ (2)

**Fig 9:**
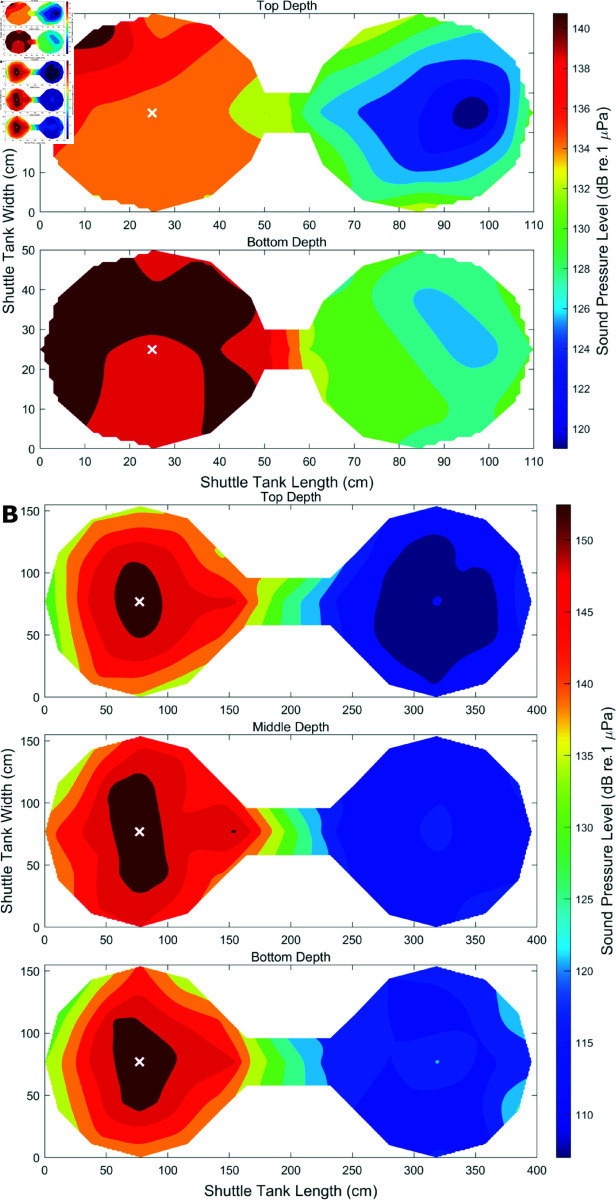
Sound pressure level sound maps. Median RMS SPL (dB re. 1 μPa @ 50 - 5 kHz) are plotted at 33 points during sound presentation in the left chamber of the A) small shuttle box, B) and 53 points in the large shuttle tank. SPL was measured at two depths in the small tank, and three depths in the large. A total of 225 hydrophone locations were used. The active speaker is indicated with a white X.

**Fig 10:**
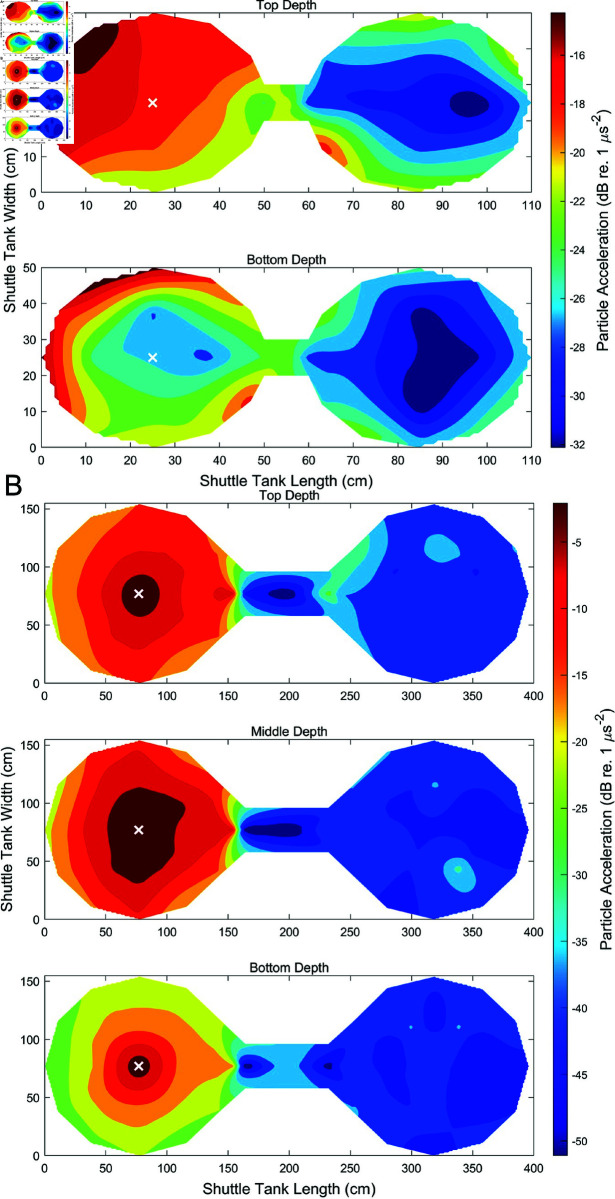
Particle acceleration sound maps. PA (dB re. $1\;{ms}^{-2}$) are plotted at 33 points during sound presentation in the left chamber of the A) small shuttle box, B) and 53 points in the large shuttle tank. PA was measured at two depths in the small tank, and three depths in the large. A total of 225 hydrophone locations were used. The active speaker is indicated with a white X.

In the small tank, 33 points were sampled at two depths, 5 cm from the water surface and 5 cm from the bottom of the tank. In the large tank, measurements were taken at 53 points at three depths: 15 cm below the water surface, in the middle of the water column, and 15 cm above the marble gravel at the tank bottom. Once the speaker in one chamber was activated, the broadband sound created one chamber with relatively high SPL and PA, a gradient of sound across the connecting column, and a sound refuge in the opposite chamber (Fig 9 & 10). In the small tank, PA ranged from -37 dB (re. $1\;{ms}^{-2}$) (19 dB above ambient) to -12 dB (42 dB above ambient), while SPL ranged from 119 dB (re. 1 μPa) (32 dB above ambient) to 143 dB (50 dB above ambient). In the large tank, PA ranged from -47 dB (1 dB above ambient) to 3 dB (50 dB above ambient), while SPL ranged from 107 dB (16 dB above ambient) to 157 dB (56 dB above ambient). Constant monitoring of the sound was not necessary as SPL was calibrated prior to each experiment, and was verified during sound mapping. Sound maps were generated using SPL values in MATLAB by interpolating between hydrophone locations and applying a contour at each depth.

### ${CO}_{2}$ threshold determination

**Fig 11:**
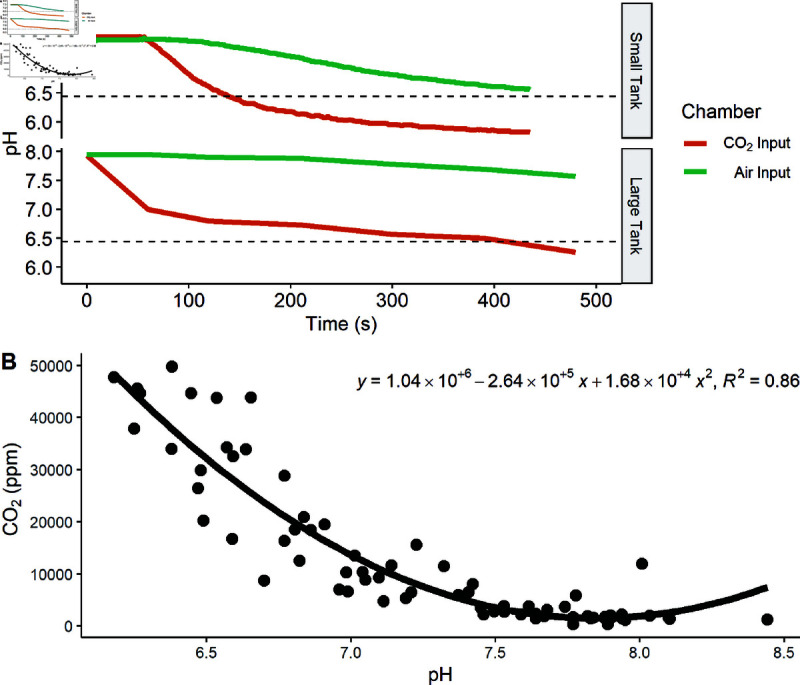
A. Water pH is plotted versus time (seconds) in the large and small shuttle tanks. Time zero represents ${CO}_{2}$ bubble initiation in the experimental chamber. The dotted line indicates the approximate threshold for ${CO}_{2}$ avoidance in bigheaded carps. **B. Model to predict p${CO}_{2}$ from pH in the large tank.** Dissolved ${CO}_{2}$ is plotted versus pH in the large tank. Water samples were collected from the increasing p${CO}_{2}$ chamber after first exit and three minutes after the last exit. The solid line represents the model described by the equation above (n = 72, F = 209, p < 0.0001, adj $R^{2}=0.854$). Water pH was used as a proxy to determine ${CO}_{2}$ concentrations according to the model in the large tank.

Carbon dioxide has been approved as a pesticide to control bigheaded carps, although the threshold for behavioral modification must be determined [49]. The ${CO}_{2}$ concentration necessary for carp movement to the opposite side of the small shuttle tank was determined by placing a school of small bighead (n = 5 schools; n = 5 individuals per school) or silver (n = 5 schools; n = 5 individuals per school) carp in the channel between the two chambers and allowing them to acclimate for 30 minutes at ambient temperature and dissolved ${CO}_{2}$ concentration In the large tank, schools of small silver carp (n = 10 schools; n = 5 individuals per school), or individual large bighead carp (n = 10 individuals) were used. The pH was 7.63 ± 0.14, total alkalinity (TA) was 167 ± 2 ppm, and water temperature was 20.6 ± 0.1 °C for the small tank trials and the pH was 7.84 ± 0.03, TA was 242 ± 2 ppm, and water temperature was 17.3 ± 0.2 °C for the larger tank trials. Compressed air was bubbled at a consistent rate into both chambers during the acclimation period and then one randomly selected chamber was switched from receiving air to ${CO}_{2}$ gas with the opposite chamber continuing to bubble air as a control (Fig 11A). The choice tank and buffer columns were drained after each trial and tanks were refilled with freshwater. Total alkalinity was measured with a colorimeter prior to injecting ${CO}_{2}$ (HI755 Checker HC; Hanna Instruments; Woonsocket, RI). Atmospheric ${CO}_{2}$ levels were continuously monitored with a tabletop sensor (Model FD-${CO}_{2}$000-USB; Forensic Detectors; Rolling Hills Estates, CA) for researcher safety.

In the small tank, ${CO}_{2}$ was bubbled into the buffer column supplying the chamber containing the school while the opposite (control) chamber received aerated water. The pH in both chambers was monitored with Sentix pH probes (Xylem Analytics) and recorded every 1 s with WTW 3310 pH meters (Xylem Analytics). Instantaneous dissolved ${CO}_{2}$ was calculated with the relationship between pH, TA, and ${CO}_{2}$ in ${CO}_{2}$Calc [50]. Schools (n=5) of bighead and silver (n=5 schools) carp were tested to determine the average ${CO}_{2}$ concentration necessary to flight-response. As the carp moved between the chambers repeatedly throughout the trial, a flight-response was defined as the last time at least three of the five fish (the majority) moved to the opposite chamber. At this point, 100 mL water samples were collected from both buffer columns in Nalgene bottles. As a secondary measurement of dissolved carbon dioxide obtained from ${CO}_{2}$Calc, ${CO}_{2}$ was measured directly from these samples with an NDIR gas analyzer and ${CO}_{2}$ probe (GMP221 20% ${CO}_{2}$ Probe; Vaisala; Vantaa, Finland) [51]. The ${CO}_{2}$ probe was sealed in a waterproof PTFE sleeve (International Polymer Engineering; Tempe, AZ) with PlasiDip (Plasti Dip International; Blaine, MN) and allowed to equilibrate with the water sample for 30 minutes before recording the ${CO}_{2}$ in parts per million (ppm) [52].

In the large tank, when the individual bighead carp (n = 10 schools) or the majority (3 or more) of the school of silver carp (n = 9 schools; n = 5 individuals per school) moved away from the ${CO}_{2}$ chamber and towards the air chamber for the first time, a 100 mL water sample was collected carefully from the ${CO}_{2}$ chamber to avoid disturbing the fish on the opposite end of the tank. A pH probe (Xylem Analytics) was placed in each chamber. Throughout the trial, fish position and pH were manually recorded. The trial continued with the fish freely moving between the two chambers until the individual stayed in the control chamber for at least three minutes. At the end of the trial, a 100 mL water sample was collected, and the final pH recorded. The fish were removed from the experimental tank and returned to the holding tanks to recover. The shuttle tank was drained while air was bubbled into both chambers to strip ${CO}_{2}$ from residual water in the marble gravel, then refilled with freshwater for subsequent trials. The relationship between instantaneous pH and dissolved ${CO}_{2}$ was calculated to estimate ${CO}_{2}$ during avoidance (F = 209, p < 0.0001, adj $R^{2}=0.854$, Fig 11B). Dissolved ${CO}_{2}$ can be accurately predicted by pH because the water temperature and TA is relatively consistent between trials in the large tank [53].

### Conditioning

In the small tank, bighead (n = 5 schools; n = 5 individuals per school) or silver carp (n = 5 schools; n = 5 individuals per school) were added to one randomly selected chamber of the choice tank with the shuttle door closed and allowed to acclimate for 30 minutes. An equal number of schools (n=5 for each species) served as non-conditioned controls that were not subjected to ${CO}_{2}$ treatment. Immediately after the acclimation period, broadband sound was played at 145 dB (re. 1 μPa) (0.06 – 5 kHz, S1) simultaneously with the start of ${CO}_{2}$ bubbling. Fish were exposed to both stimuli concurrently for four five-minute treatments daily over two days. Carbon dioxide was bubbled into the side of the experimental tank containing the fish until reaching the threshold established in the ${CO}_{2}$ avoidance experiment, as determined by continuous measurement of pH. After the five-minute conditioning treatment, fish were moved to the opposite chamber containing aerated freshwater and allowed to recover for 15 minutes while the conditioning chamber was drained and refilled. After the recovery period, ${CO}_{2}$ and sound conditioning treatments were repeated a total of four times each day. Schools were presented paired acoustic and ${CO}_{2}$ stimuli for two consecutive days.

In the large tank, individual bighead carp were conditioned with air and broadband sound as a control (n=10), or with sound and elevated ${CO}_{2}$ (n=10). Carp were exposed to either ${CO}_{2}$ and sound or compressed air and sound stimuli for four ten-minute treatments daily over two days. Individuals were added to one randomly selected chamber of the tank with the channel blocked by clear plexiglass and allowed to acclimate for 30 minutes. Then, the assigned gas was bubbled into the experimental tank. Carp remained in the blocked chamber and the broadband sound was played at 155 dB (re. 1 μPa) (S2) simultaneously with the addition of gas bubbles to threshold levels for ten minutes. After the first ten-minute conditioning treatment, fish were moved to the opposite chamber and allowed to recover for 15 minutes before the second treatment. Before the third treatment, fish were removed from the experimental tank and allowed to recover in the holding tank for one hour. Meanwhile, the entire experimental tank was drained and refilled with freshwater for the two remaining conditioning treatments. Fish were presented paired stimuli four times daily over two consecutive days.

### Negative phonotaxis response behavior

In the small tank, the day following the last conditioning treatment, individual schools were placed in a randomly selected chamber and allowed to acclimate for 30 minutes. After the acclimation period, broadband sound was played at 145 dB (re. 1 μPa) for five minutes from one randomly selected speaker while only aerated water recirculated through the tanks. Conditioned and unconditioned schools were tested separately, but on the same daily schedule and in the same water conditions. Water temperature was consistent across conditioning treatments, at 17.6 ± 1.0 °C for the control and 16.1 ± 0.6 °C for the ${CO}_{2}$ conditioned fish. All schools were tested on day 1, 4, 7, 11, 14, 21 and 28 after conditioning.

Individual adult bighead carp were placed in the shuttle of the large tank the day after the last conditioning treatment and allowed to acclimate for 30 minutes. After the acclimation period, sound (155 dB re. 1 μPa) onset began once the fish entered a reaction zone, defined as the sector of the circular chamber between the speaker and two walls of the exit. Sound was played for five minutes. Water temperature was consistent across conditioning treatments, at 17.6 ± 0.5 °C for the control and 17.5 ± 0.5 °C for the ${CO}_{2}$ conditioned fish. Fish were tested three times each day on day 1, 3 and 7 after conditioning.

### Behavioral analysis

Behavior was assessed from overhead videos (25 frames per second) played back using VLC player (Version 3.0.18). In all behavior trials, the last 15 minutes of the 30-minute acclimation period were filmed to assess control swimming behavior, this is referred to as the “pre-sound” period. For both the small and large tank experiments, negative phonotaxis behavior was evaluated as the time to exit the sound chamber and fully enter the opposite choice chamber during sound playback, and the time spent in the opposite chamber before returning to the sound chamber. Negative phonotaxis was defined by the majority of the school (3 or more fish in the small tank), or the single bighead (large tank only), moving their entire body out of the connecting shuttle and into the opposite circular chamber. In the large tank, tracking software (Ethovision XT Version 11.5.1026; Noldus; Wageningen, the Netherlands) was used to quantify additional behavioral metrics including stationary time and distance from the fish to the active speaker. Fish were considered exhibiting freeze-responses when their location changed less than Â1/4 of body length every three seconds. To quantify flight-responses, fish distance to the speaker was averaged over the five minute duration of the trial. Control swimming during the pre-sound period was evaluated for time to exit the sound chamber and fish distance to the speaker for the final five minutes of the pre-sound period.

### Statistical methods

Separate mixed ANOVAs were run using time to exit and return to the sound chamber as response variables with schools where appropriate as the experimental unit. Mixed ANOVA allows for the evaluation of between-subjects factors, such as conditioning treatment, as well as within-subjects factors, such as time since conditioning. The presence of the sound stimulus, conditioning treatment, and the day since trial onset and their interactions were used as predictors, as well as either individual fish ID or school ID as a random effect. The assumptions of no outliers, normality of residuals, homogeneity of variances and covariances were met unless otherwise noted. In the small tank, Welch’s Two-Sample T-tests to account for unequal variances were performed to compare negative phonotaxis responses between conditioned and unconditioned schools for each day. In the large tank, paired T-tests were performed to compare behavior before and during sound playback for each fish. A significance threshold of α = 0.05 was set a priori as per convention. All statistical analysis was performed in R version 4.1.0, and the dataset and reproducible code are hosted publicly [48].

## Supporting information

S1 Fig Small tank spectrogram and power spectrum. Upper: Spectrogram and Lower: power spectrum. Hydrophone recording of the broadband sound stimulus played in the small choice tank for conditioning and phonotaxis experiments. Recording was taken in the middle of the water column, between the speaker and the shuttle exit. (TIFF)

S2 Fig Large tank spectrogram and power spectrum. Upper: Spectrogram and Lower: power spectrum. Hydrophone recording of the broadband sound stimulus played in the large choice tank for conditioning and phonotaxis experiments. Recorded in the middle of the water column directly above the speaker. (TIFF)

S3 Fig Experimental setup. A. The small shuttle tank setup, including solenoids to control flow of air and ${CO}_{2}$ into the mixing buffer columns, recirculation pumps, and pH meters. B. Large tank setup, including underwater speaker placement. C. Overhead view of the small tank setup, with two speakers and a school of five silver carp on the left side. D. Overhead view of the large tank setup, with two speakers and an individual bighead carp on the left side. (TIFF)
