# Supplementary figures and images for: Conditioning invasive bigheaded carps (Hypophthalmichthys molitrix and H. nobilis)to enhance the efficacy of acoustic and CO2 deterrents
Source: PLoS One. 2025 May 19;20(5):e0320395. doi: 10.1371/journal.pone.0320395 (PMC12088057; doi:10.1371/journal.pone.0320395)

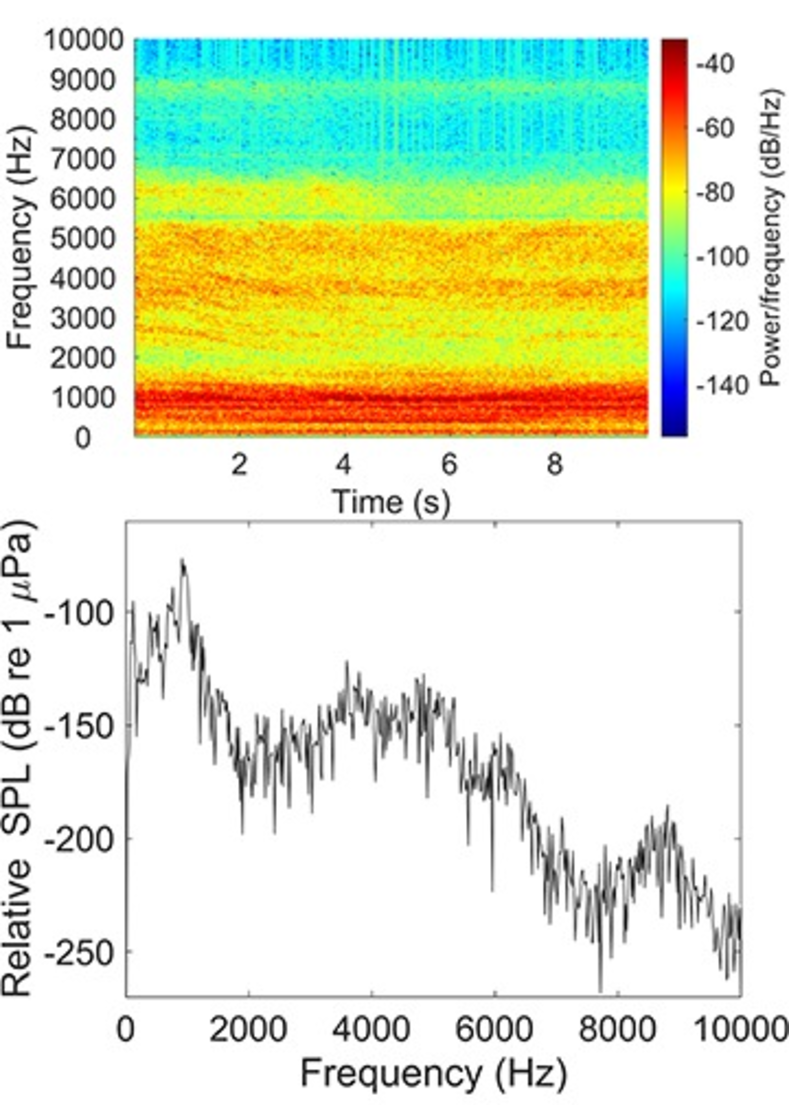

Supplement: S1 Fig — Upper: Spectrogram and Lower: power spectrum. Hydrophone recording of the broadband sound stimulus played in the small choice tank for conditioning and phonotaxis experiments. Recording was taken in the middle of the water column, between the speaker and the shuttle exit. (TIFF) [file pone.0320395.s001.tif]

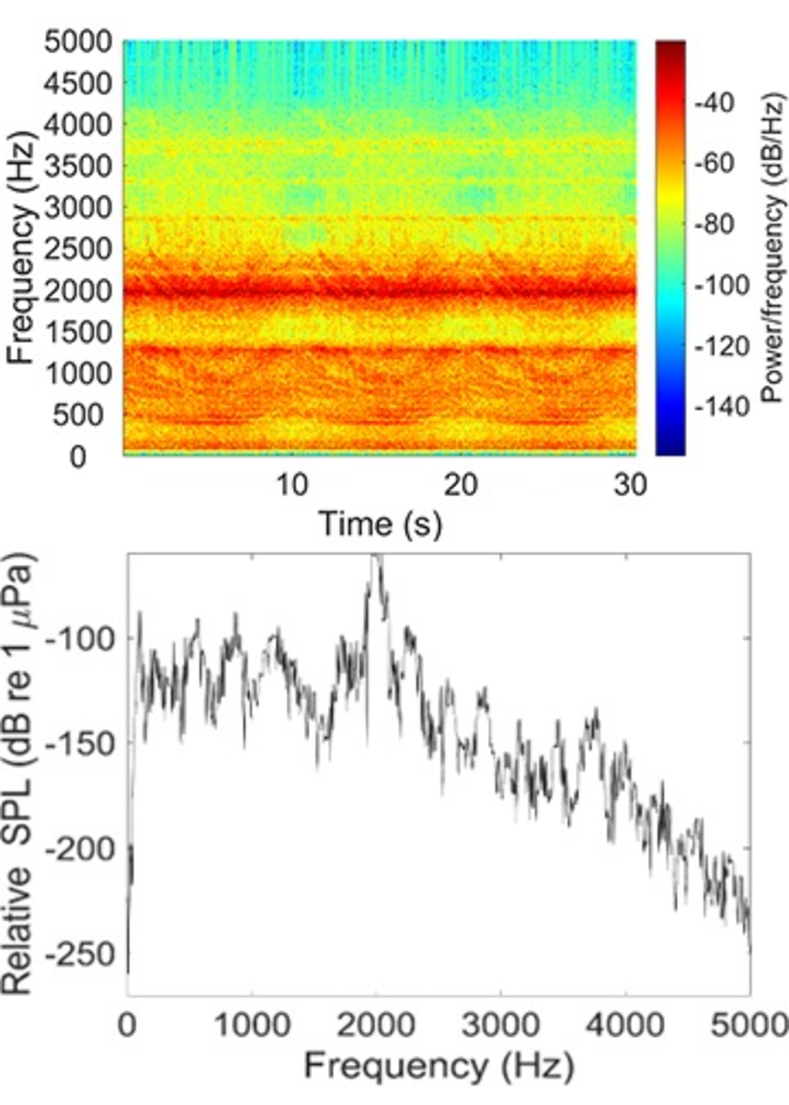

Supplement: S2 Fig — Upper: Spectrogram and Lower: power spectrum. Hydrophone recording of the broadband sound stimulus played in the large choice tank for conditioning and phonotaxis experiments. Recorded in the middle of the water column directly above the speaker. (TIFF) [file pone.0320395.s002.tif]

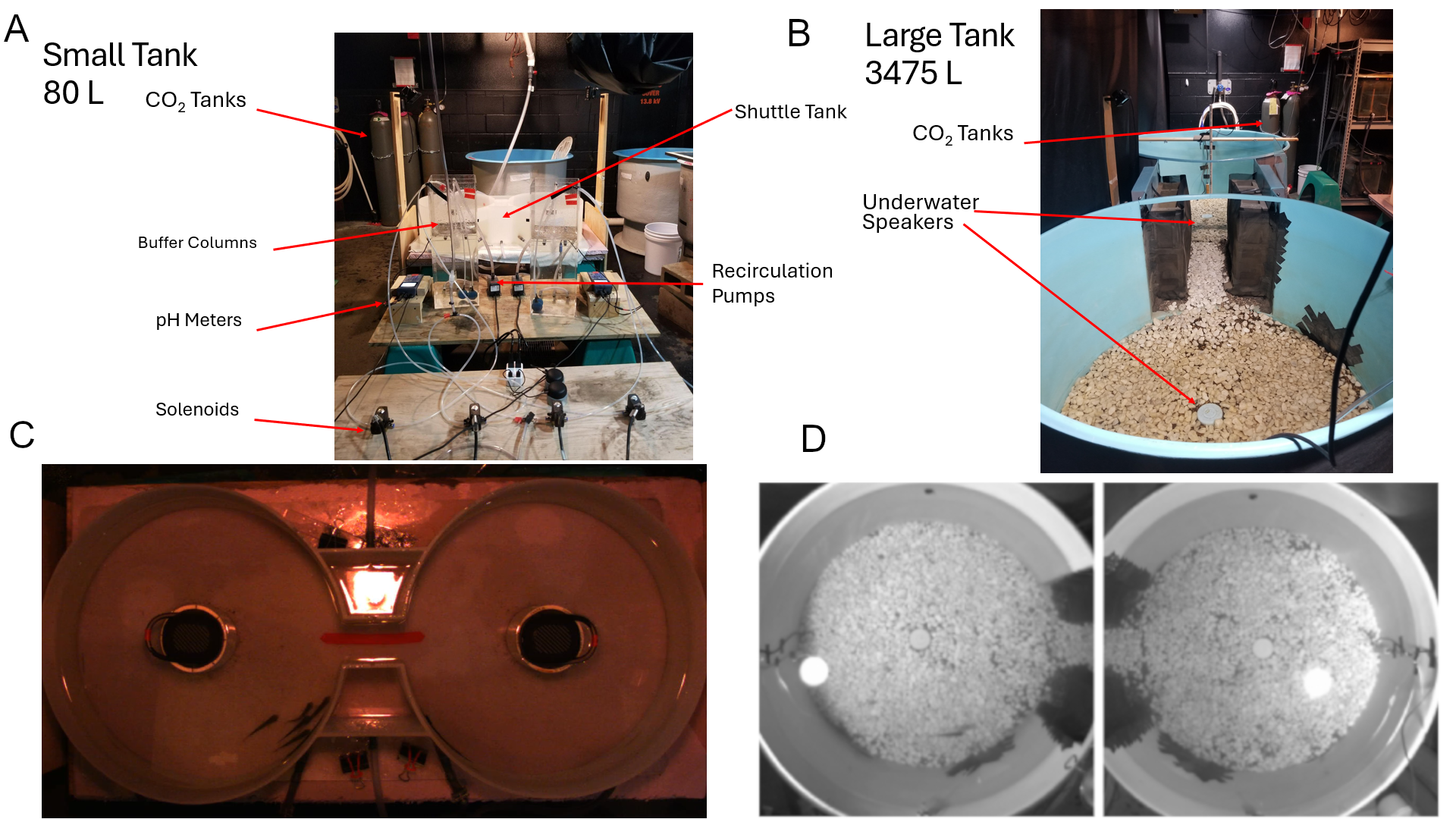

Supplement: S3 Fig — A. The small shuttle tank setup, including solenoids to control flow of air and CO2 into the mixing buffer columns, recirculation pumps, and pH meters. B. Large tank setup, including underwater speaker placement. C. Overhead view of the small tank setup, with two speakers and a school of five silver carp on the left side. D. Overhead view of the large tank setup, with two speakers and an individual bighead carp on the left side. (TIFF) [file pone.0320395.s003.tif]
